# Allergic Bronchopulmonary Aspergillosis Mimicking Lung Cancer: A Case Report

**DOI:** 10.7759/cureus.43632

**Published:** 2023-08-17

**Authors:** Alejandro J Torre-De León, Herik Valles-Bastidas, Horiana B Grosu

**Affiliations:** 1 School of Medicine and Health Sciences, Tecnologico de Monterrey, Monterrey, MEX; 2 Pulmonary Medicine, MD Anderson Cancer Center, Houston, USA

**Keywords:** allergic bronchopulmonary aspergillosis, hypersensitivity, asthma, lung cancer, pulmonary masses

## Abstract

Patients with allergic bronchopulmonary aspergillosis (ABPA), a hypersensitivity reaction to *Aspergillus fumigatus*, typically present with asthma; the common imaging findings are central bronchiectasis, mucoid impaction, and tree-in-bud opacities. In this report, we discuss the case of a heavy smoker who presented with a large pulmonary mass that was initially presumed to be primary lung cancer and who was ultimately diagnosed with ABPA, which responded favorably to steroid treatment.

## Introduction

Allergic bronchopulmonary aspergillosis (ABPA) is a complex immunological pulmonary disorder caused by a hypersensitivity reaction to *Aspergillus fumigatus*, a common indoor and outdoor mold [1-5]. *Aspergillus* can cause various diseases including pulmonary aspergillosis, rhinosinusitis, central nervous system aspergillosis, endocarditis, osteomyelitis, and endophthalmitis. *Aspergillus fumigatus*, the primary causative agent of aspergillosis, accounts for 90% of human infections [6]. ABPA is the most severe form of *Aspergillus* infection among atopic patients. The estimated prevalence of ABPA among people with asthma is 1-3.5% [3]. The characteristic symptoms of ABPA are low-grade chronic fever, coughing, wheezing, chest pain, and fleeting pulmonary opacities. Malaise, weight loss, and hemoptysis may also be present, and patients may expectorate sputum containing brown mucus and plugs [1-3]. However, some patients with ABPA are asymptomatic initially or have a cough attributed to a different etiology, such as smoking [2]. Common features on high-resolution CT include central bronchiectasis, mucoid impaction, mosaic attenuation, centrilobular nodules, and tree-in-bud opacities [1,4]. ABPA is underdiagnosed, and the average time from symptom onset to diagnosis may be as high as 10 years [2]. Unrecognized ABPA could lead to bronchiectasis and subsequent respiratory failure [1,3]. In this report, we present a case of ABPA mimicking lung cancer. This case report highlights the complexity of this underdiagnosed disease, which should be kept in mind as a possible cause of unexplained respiratory symptoms.

## Case presentation

**Figure 1:**
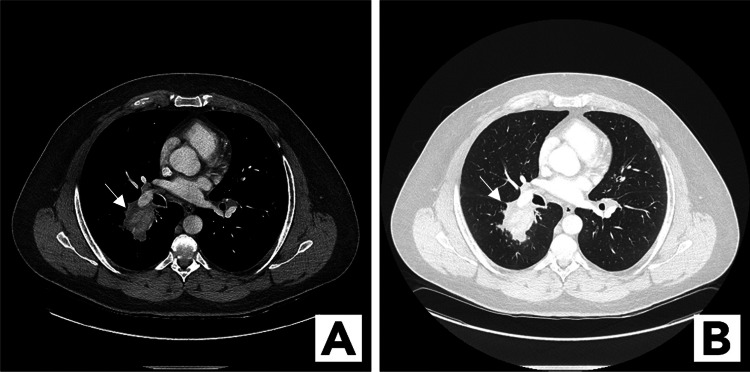
Hilar mass Mediastinal window (A) and lung window (B) CT revealed a solitary 57 x 48-mm hilar mass (arrows) associated with hilar adenopathy CT: computed tomography

A 55-year-old man, a heavy smoker, presented for evaluation after he had developed shortness of breath and severe fatigue. The patient had a medical history of poorly controlled asthma since childhood and coronary artery disease. As part of the workup for his shortness of breath, the patient underwent a chest CT, which showed a solitary 57 x 48-mm right hilar mass associated with right hilar adenopathy (Figures 1A, 1B). Given this finding and the patient’s risk factors for lung cancer, there was a high suspicion of primary lung cancer. A diagnostic bronchoscopy with endobronchial ultrasonography (EBUS) and transbronchial needle aspiration (TBNA) was performed. Both the mass and lymph node were biopsied via EBUS-TBNA. Cytologic analysis of lymph node biopsy samples showed normal lymphoid tissue and cytologic analysis of mass biopsy samples showed reactive epithelial cells in a background of debris and acute inflammation. A complete pulmonary function test demonstrated moderate airflow obstruction with significant bronchodilator response and normal diffusing capacity of the lungs for carbon monoxide. The patient developed significant wheezing after bronchoscopy and was given budesonide and formoterol for presumed asthma exacerbation, in addition to a course of prednisone. Despite the negative bronchoscopy biopsy results, the patient was also referred for a percutaneous biopsy, as the pretest probability of malignancy remained high. During the limited CT done prior to percutaneous biopsy at two weeks post bronchoscopy, it was noted that the mass had shrunk significantly, and lung biopsy was deemed unnecessary. This was attributed to the patient being on prednisone.

**Figure 2:**
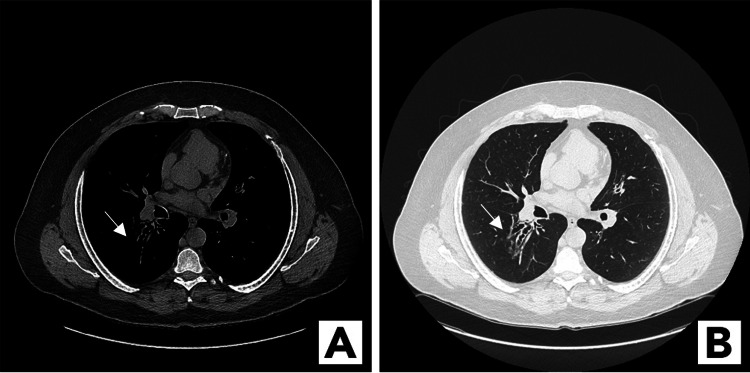
Bronchiectasis Mediastinal window (A) and lung window (B) CT showed hyperdense tubular opacities and dilated bronchi with inspissated mucus, consistent with bronchiectasis (arrows) CT: computed tomography

Further workup revealed a high serum level of *Aspergillus*-specific immunoglobulin E (IgE), and a review of the results of prior laboratory tests demonstrated a high eosinophil count. Based on these results, the patient was diagnosed with ABPA and treated with oral prednisone for six weeks. We offered to add itraconazole to this regimen, but the patient refused to have any monitoring of his liver function test. Repeat CT performed at the completion of this treatment showed resolution of the hilar mass as well as hyperdense tubular opacities and dilated bronchi with inspissated mucus, findings that were consistent with bronchiectasis (Figures 2A, 2B). The patient’s symptoms resolved without further treatment, and he was subsequently lost to follow-up.

## Discussion

This case highlights the importance of considering ABPA in the differential diagnosis of patients with unexplained respiratory symptoms, and it underscores the need for clinicians to have a high index of suspicion for ABPA in such patients to recognize the disease promptly.

The diagnostic challenges associated with ABPA are evident in the present case. Since radiological findings showed a high-density round right hilar mass with thickened mucus in dilated bronchi secondary to obstruction, we initially suspected lung cancer, which emphasizes the need to use caution and explore complementary possibilities and differential diagnosis. It should be noted that aspergillosis should be considered in these patients if the above-mentioned radiological presentation or symptomatology is seen. Radiological findings that are highly suggestive of ABPA include bronchiectasis affecting three or more lobes; centrilobular nodules; and mucoid impaction [1,3,4]. Familiarity with these characteristic imaging findings can aid in differentiating ABPA from other pulmonary disorders. ABPA should be distinguished from pulmonary aspergillosis, which can cause an air crescent sign when as aspergilloma forms in the already-existing lung cavity, or from invasive aspergillosis during the recovery phase when air fills the space between the parenchyma and retracting devitalized infected lung tissue. The air crescent sign is not specific and can be seen in other infections as well, such as melioidosis [7].

Large pulmonary masses are a distinctly unusual manifestation of ABPA. Agarwal et al. have reported the cases of three patients with pulmonary masses and ABPA, each due to a different mechanism [8]. Pulmonary masses are typically caused by mucus plugging in the bronchi and distal secretion buildup. Alternatively, they may result from large bronchoceles (mucus-filled dilated bronchi), without proximal obstruction. Another possible mechanism is inflammatory eosinophilic parenchymal consolidation, presenting as a pseudotumor without endobronchial involvement, as seen in our patient [8,9].

ABPA has various immunologic features, including immediate hypersensitivity (type I), antigen-antibody complexes (type III), and eosinophil-rich inflammatory cell responses (type IVb). All patients with asthma should be routinely screened for *Aspergillus* with skin tests [1]. If asthma is considered essential for the recognition of the disease, the diagnosis will be missed [10]. In the absence of asthma, ABPA is often not included as a top differential diagnosis item as other pulmonary disorders, such as lung cancer or pulmonary tuberculosis [1,11]. There are certain criteria for the diagnosis of ABPA. The Rosenberg-Patterson criteria, comprising eight major and three minor components, served as the predominant method for diagnosing ABPA in asthma. However, there was no consensus on the required number of criteria for a diagnosis, and the specific threshold for various immunological tests was not defined [12]. To address this matter, the International Society for Human and Animal Mycology (ISHAM) allergic bronchopulmonary aspergillosis (ABPA) working group (AWG) devised a novel set of criteria to accurately diagnose ABPA in individuals with asthma. These new criteria encompass up to five elements: the presence of both (1) serum Af-specific IgE >0.35 kUA/L and (2) serum total IgE > 500 IU/mL, and at least two of the following features: (1) serum Af-specific IgG >27 mgA/L; (2) bronchiectasis on CT chest; (3) peripheral blood total eosinophil count (TEC) >500 cells/μL [12,13]. In addition, ISHAM classified ABPA into five stages. The prognosis in patients with stage I-IV is excellent and most achieve complete remission. Patients with stage V are more likely to develop pulmonary fibrosis and even irreversible lung injury [12,13].

The first-line therapy for ABPA is treatment with oral corticosteroids [1,3] to decrease serum IgE levels by 35-50%, leading to clinical and radiological improvement; during follow-up, a doubling of the baseline IgE level denotes an ABPA relapse [1]. After six weeks of prednisone treatment, our patient’s symptoms improved, and he was lost to follow-up.

## Conclusions

Clinicians should be aware that ABPA can present as a solitary lung mass, mimicking lung cancer. Oral corticosteroids continue to be the main therapy for ABPA. Early diagnosis and treatment of ABPA can prevent disease progression to an irreversible lung injury.
